# Iron-Induced Lipid Oxidation Alters Membrane Mechanics Favoring Permeabilization

**DOI:** 10.1021/acs.langmuir.4c03294

**Published:** 2024-11-12

**Authors:** Sara Lotfipour Nasudivar, Lohans Pedrera, Ana J. García-Sáez

**Affiliations:** † Institute for Genetics and Cologne Excellence Cluster on Cellular Stress Responses in Aging-Associated Diseases (CECAD), University of Cologne, Joseph-Stelzmann-Strasse 26, 50931, Cologne, Germany; ‡ Max Plank Institute of Biophysics, Max-von-Laue-Straße 3, 60438, Frankfurt am Main, Germany

## Abstract

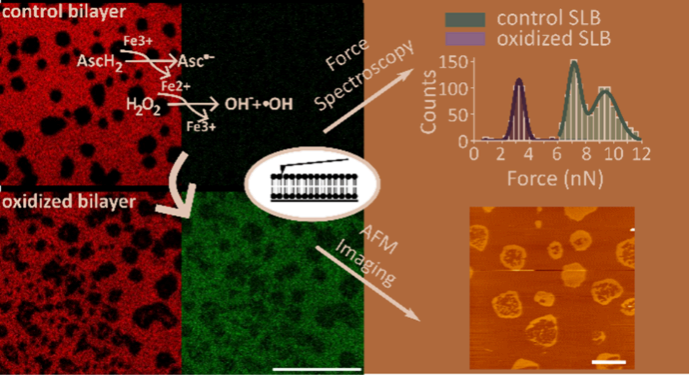

Ferroptosis is a form of regulated necrosis characterized by the iron-dependent accumulation of lipid peroxides in cell membranes. However, how lipid oxidation via iron-mediated Fenton reactions affects the biophysical properties of cellular membranes and how these changes contribute to the opening of plasma membrane pores are major questions in the field. Here, we characterized the dynamics of membrane alterations during lipid oxidation induced onsite by Fenton reactions in chemically defined *in vitro* model membrane systems. We find that lipid vesicle permeabilization kinetically correlates with the appearance of malondialdehyde (MDA), a product of lipid oxidation. Iron-induced lipid oxidation also alters the lateral organization of supported lipid bilayers (SLBs) with lipid phase coexistence in a time-dependent manner, reducing the lipid phase mismatch and the circularity of liquid ordered domains, which indicates a decrease in line tension at the phase boundaries. Further analysis of oxidized SLBs by force spectroscopy reveals a significant decrease in the average membrane breakthrough force upon oxidation, resulting from changes in lipid bilayer organization that make it more susceptible to permeabilization. Our findings suggest that lipid oxidation via iron-mediated Fenton-like reactions induces strong changes in membrane lipid interactions and mechanical properties leading to reduced line tension in the permeabilized state of the bilayer, which promotes membrane pore formation.

## Introduction

The plasma membrane acts as a permeability barrier that separates the inside from the outside of the cell, thus defining the cell and limiting the transport of molecules. It consists of a closed bilayer of amphiphilic lipids with embedded proteins that together determine its physical properties.^1^ Retaining plasma membrane integrity is critical for cellular function and several forms of regulated cell death culminate with plasma membrane disruption. Among them, ferroptosis is a caspase-independent form of regulated necrosis characterized by the accumulation of iron-dependent lipid peroxides within cellular membranes.^2^ In contrast to other types of regulated cell death, which possess a specific protein machinery to mediate plasma membrane disruption, no protein executors have been identified that mediate plasma membrane permeabilization or pore formation in ferroptosis. Instead, ferroptosis is induced when lipid oxidation overwhelms cellular antioxidant defenses.^2,3^ Ferroptotic cells are characterized by plasma membrane rupture and alteration of membrane phospholipids properties.^2^ Among them, the formation of nanopores at the plasma membrane has emerged as a central mechanism for the execution of the final step of this type of cell death.^4^

Recent studies have suggested that nonenzymatic lipid peroxidation by Fenton-like reactions is the predominant mechanism driving membrane oxidation during ferroptosis.^5,6^ In its simplest form, the Fenton reaction (H_2_O_2_ + Fe^2+^ → Fe^3+^ + ^•^OH + HO^–^) describes the reaction between Fe^2+^ and H_2_O_2_ to form a hydroxyl radical (^•^OH) and a hydroxyl anion.^7,8^ In the cellular environment, the hydroxyl radical initiates lipid peroxidation in a chain reaction that depends on the presence of polyunsaturated fatty acids in the membrane phospholipids and on oxygen concentration, resulting in the formation of multiple lipid products including peroxidized lipids and their derivatives. Indeed, peroxidized lipids tend to hydrolyze into oxidatively truncated phospholipids with shorter acyl chains which play a key role in membrane permeabilization and ferroptosis execution.^9^ However, the role of lipid oxidation via Fenton-like reactions in the alteration of the biophysical and mechanical properties of membranes that are required for plasma membrane permeabilization in ferroptosis remains poorly understood.

The effect of oxidized lipids in simplified model membrane systems has been extensively studied over the past decades because oxidative stress-related diseases are associated with altered cellular membranes. The presence of oxidized lipids has been shown to induce major changes in membrane properties, including increases in the area occupied by lipids^10−14^ and in membrane fluidity,^15−18^ changes in phase behavior and domain formation,^10,13,19−23^ as well as increased permeability and pore formation.^10,24−29^ These changes in membrane properties were dependent on the membrane lipid composition. In this regard, phosphatidylethanolamine (PE) oxidation has been reported to stabilize a lamellar system through the formation of Schiff bases between PE head groups and aldehyde species^20^ presumably generated from oxidatively truncated phospholipids.^30^ Truncated lipids with a terminal carboxyl group in the oxidized tail have also been associated with an increase in membrane fluidity due to their tendency to orient toward the aqueous solution.^15^ Dynamic simulation studies have proposed that the increase in membrane permeability under lipid oxidation is related to the tendency of oxidized lipids, especially those with a shorter tail, to bend toward the polar interface where oxygen atoms form hydrogen bonds with water and the polar lipid headgroups.^10,25,27^ However, it was not always possible to dissect which lipid modification is responsible for specific changes in membrane properties. Despite the variety of membrane alterations induced by oxidized lipids reported in the literature, we fail to understand how the membrane mechanical alterations induced by lipid oxidation are connected to the initiation of membrane breakdown. In addition, in the context of ferroptosis, little is known about the membrane changes and their dynamics specifically caused by lipid oxidation via Fenton-like reactions and how they are connected to the plasma membrane breakdown observed in ferroptotic cells.

Here, we investigated membrane alterations induced over time by onsite lipid oxidation via Fenton-like reactions in two *in vitro* model membrane systems using lipid vesicles and SLB. By reducing the complexity of the cellular context, lipid model membranes have the advantage of being chemically controlled systems that enable the study of fundamental changes in lipid bilayer organization upon lipid oxidation. Using a liposome leakage assay, we find that vesicle permeabilization kinetically correlates with the appearance of malondialdehyde (MDA), a product of lipid oxidation. We also show that lipid oxidation dynamically alters the lateral organization of membranes with coexistence of liquid ordered (Lo) and liquid disordered (Ld) phases, leading to changes in topography and domain shape associated with a decrease in the line tension between phases. Finally, we associate membrane remodelling upon oxidation with a decrease in the average breakthrough force of the membrane. Our results support a general role for lipid oxidation as a promoter of strong changes in the mechanical properties of membranes, which are relevant for membrane pore formation and plasma membrane disruption in ferroptosis.

## Experimental Section

### Materials

All reagents and lipid standards were purchased from Sigma-Aldrich (St. Louis, USA) or Avanti Polar Lipids (Alabaster, USA). Organic solvents were purchased from Supelco/Merck KGaA (Darmstadt, Germany). The oxidation of the liposomes was induced by the addition of various concentrations of FeSO_4_ and ascorbic acid and incubated at 37 °C at the indicated times.

### Methods

#### Permeabiliziation Assay of Large Unilamellar Vesicles

l-α-Phosphatidylcholine from egg yolk (egg-PC) was resuspended and mixed with carboxyfluorescein (CF) solution (80 mM, pH 7.0) to a concentration of 5 mg/mL. After six cycles of freezing and thawing, the lipid mixture was extruded by 31 passes through a 100 nm pore size membrane. A Sephadex G50 column was used to separate liposomes with encapsulated CF from the nonencapsulated CF using as outside buffer 300 mM NaCl, 20 mM Hepes pH7.0. A ratio of fluorescence between permeabilized and nonpermeabilized vesicles greater than 3 was used. Treatment of the vesicles with iron and ascorbate at different concentrations initiates a Fenton reaction and leads to lipid peroxidation of the phospholipids. The fluorescence signal corresponding to CF was measured every 10 min with a plate reader (Perkins) using an excitation wavelength of 485 nm and an emission wavelength of 525 nm. We used Triton X-100 to induce the total permeabilization of the vesicles in the assay. In parallel, we measured the fluorescence of untreated vesicles as a reference for nonpermeabilized vesicles. The percentage of permeabilized vesicles was calculated as follows:
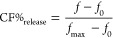


#### Measurement of Malondialdehyde with the Thioarbituric Acid Assay

The thiobarbituric acid (TBA) assay measures the reaction product of malondialdehyde (MDA) and TBA and can be used as a reference for the level of oxidation. MDA is a secondary oxidation product formed during lipid peroxidation. Two molecules of TBA form a chromogen that allows the spectrophotometric determination at 532 nm.^31^

#### Supported Lipid Bilayer

SLBs were prepared for AFM measurements and confocal microscopy. Lipid compositions were prepared and stored as a dry film. The lipids dioleoyl-phosphatidylcholine (DOPC), sphingomyelin (SM) and cholesterol (Chol) were dissolved in chloroform. The lipid film was rehydrated in PBS buffer (2.7 mM KCl, 1.5 mM KH_2_PO_4_, 8 mM Na_2_HPO_4_, 137 mM NaCl, pH 7.2) to a final concentration of 10 mg/mL and stored at −20 °C. The lipid mixture was resuspended in 140 μL of buffer (10 mM HEPES, 150 mM NaCl, 3 mM NaN_3_, pH 7.2) and sonicated for 15 min to form SUVs. The lipid suspension was deposited to a freshly prepared mica disk on a glass slide and CaCl_2_ was added to a total concentration of 3 mM in a final volume of 500 μL. After an initial incubation of 2 min at 37 °C, followed by 10 min at 65 °C and slow cooling, floating vesicles and CaCl_2_ were washed out by adding and removing 150 μL of buffer solution 20 times. SLBs were additionally stained with Bodipy BODIPY-C11 (0.1 mol %) for the tracking of oxidation or labeled with rhodamine-PE (0.5 mol %) for FRAP experiments.

#### Confocal Microscopy

Confocal fluorescence microscopy was performed using an Olympus infinity line scanning microscope (Abberior Instruments) equipped with a UPlanX APO 60× oil/1.42 NA objective. Lo circularity  values were analyzed using ImageJ (http://imagej.nih.gov/ij/) that analyzes particle function. Photobleaching experiments were performed using 60× oil 8 × 8 μm area was bleached at nominal 100% laser transmission and a serious of 5 images, following immediate recovery was captured for a serious of 80 images (interval 1.2 s).

#### Atomic Force Microscopy - Imaging

Lipid bilayers were imaged using a JPK nanowizard (JPK Instruments, Berlin, Germany) in contact or tapping mode and V-shaped nonconducting silicon nitride cantilevers (Bruker, DNP-10) with a typical spring constant of 0.06 Newton/m. The scanning rate was set between 0.6 and 2 Hz. Images were processed by applying a degree 1 or 2 polynomial fit to the surface.

#### Atomic Force Microscopy - Force Spectroscopy

For this measurement, an area was selected on a bilayer topographical image on which a set of 10 × 10 or 14 × 14 force measurements were operated. A limit is set at which the cantilever is lowered at a constant speed. The dependence of the force as the function of height can be plotted using Python. Before generating the force histograms, a peak detection script was run in Python and given out a file with detected values.

## Results and Discussion

### Lipid Oxidation Increases the Permeability of Lipid Vesicles

Despite the rapid progress in understanding ferroptosis in recent years, how the species generated via Fenton reaction-mediated lipid oxidation affect membrane biophysical properties and how these changes contribute to membrane pore opening remain major questions in the field. The accumulation of oxidized lipid species is recognized as a hallmark of ferroptosis.^9,32,33^ It is also known that lipid peroxidation induced by oxidative stress changes membrane organization and composition, resulting in significant alterations such as increased membrane permeability and modifications in lipid packing, fluidity, and viscosity.^9,11−13,34^ To assess whether the generation of oxidized lipid species via Fenton-like reactions directly affects membrane permeability, we used a minimalist reconstituted system devoid of other cellular components and based on pure lipids and large unilamellar vesicles (LUVs) made of different compositions.

**Figure 1:**
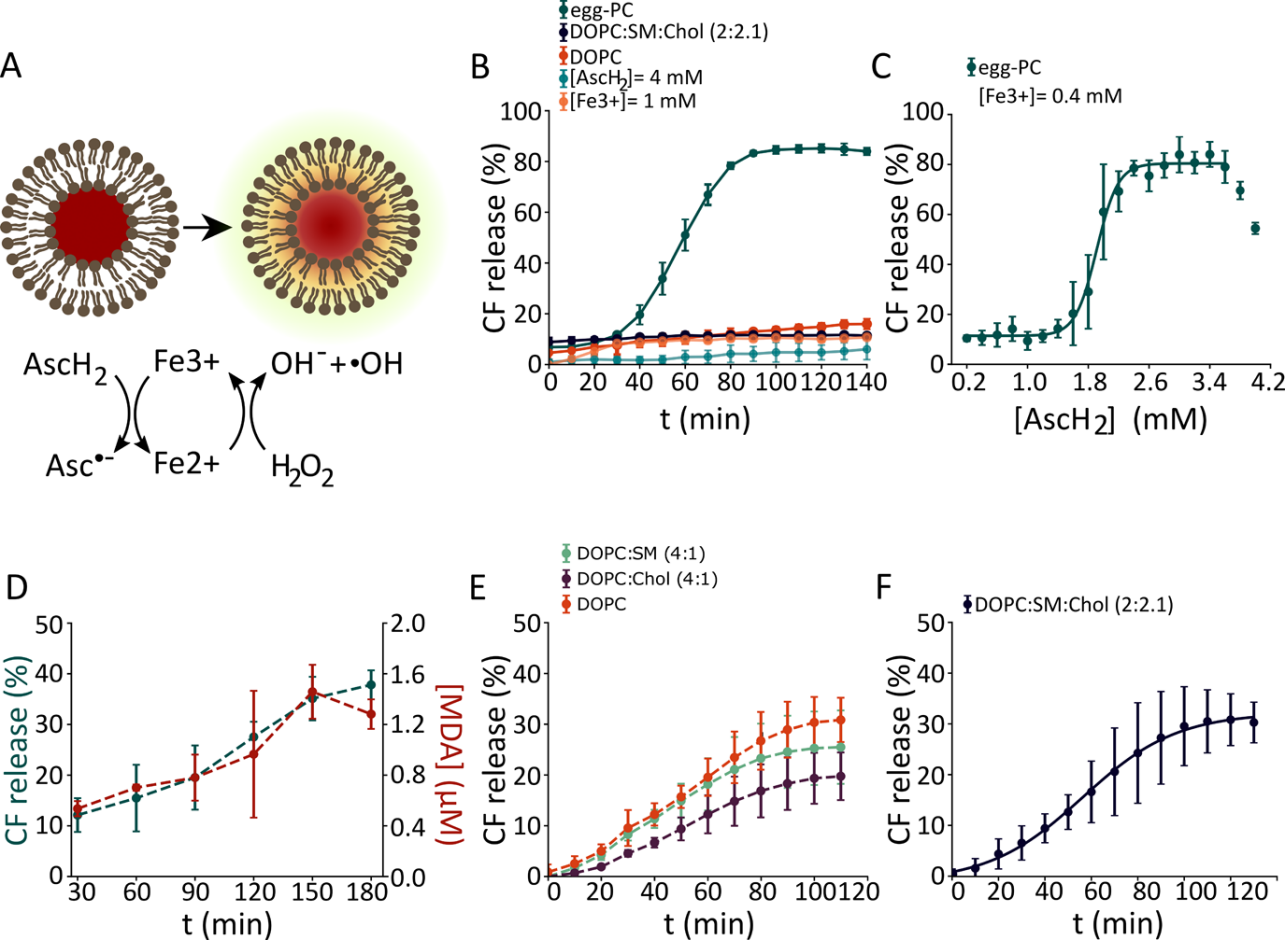
Lipid oxidation induces permeabilization of lipid vesicles. (A) Schematic representation of the experimental setup. Iron sulfate and ascorbic acid initiate Fenton-like chain reactions of lipid oxidation. Permeabilization of lipid vesicles increases the leakage of carboxyfluorescein. (B) Kinetics of carboxifluorescein leakage from liposomes made of egg-PC, DOPC, or DOPC:SM:Chol (2:2:1) upon lipid oxidation induced by 0.8 mM iron sulfate and 2 mM ascorbic acid at 37 °C. The concentrations of iron sulfate and ascorbic acid used for negative control experiments are indicated in the figure. (C) Dose–response curve of permeabilization of LUV made of egg-PC as a function of ascorbic acid concentration in the presence of 400 μM iron sulfate. (D) Kinetic curves of vesicle permeabilization and malondialdehyde concentration in egg-PC vesicles treated with 400 μM iron sulfate and 1.6 mM of ascorbic acid. (E) Kinetics of permeability increase in vesicles made of DOPC, DOPC:SM (4:1), and DOPC:Chol (4:1) upon lipid oxidation induced by 800 μM iron sulfate and 8 mM ascorbic acid. (F) Kinetics of permeabilization in vesicles made of DOPC:SM:Chol (2:2:1) treated with 8 mM ascorbic acid and 500 μM iron sulfate. All data points represent the mean values and bars indicate the standard deviations from a set of at least three independent experiments. When no error bar is observed, the corresponding standard deviation is smaller than the size of the symbol.

We initially studied egg phosphatidylcholine (PC) vesicles because they are a natural mixture of PC, a major lipid in cellular membranes, containing polyunsaturated species, required for the induction of Fenton-like reactions of lipid oxidation. We induced lipid oxidation via Fenton-like reactions onsite by adding ascorbic acid and FeSO_4_ to the vesicle mixtures (Figure 1A). Changes in vesicle permeability over time were assessed by monitoring the release of carboxyfluorescein (CF), a self-quenching dye encapsulated within the LUVs (Figure 1A). Using this liposome leakage assay, we found that, under our experimental conditions, incubation with iron and ascorbic acid caused permeabilization of LUVs made of egg-PC, starting at around 20 min and reaching saturation at approximately 80 min, whereas neither ascorbic acid nor FeSO_4_ individually were able to affect vesicle stability (Figure 1B). We also observed a dose-dependent relationship between ascorbic acid concentration and CF release from LUVs made of egg-PC, with maximal permeabilization capacity at ascorbic acid concentrations above 2.4 mM (Figure 1C).

To further characterize the temporal relationship between the extent of lipid oxidation and LUVs permeabilization, we tracked the carboxifluorescein fluorescence and lipid oxidation measured by the thiobarbituric acid reactive substance (TBARS) assay in parallel (Figure 1D). The appearance of malondialdehyde (MDA), a secondary product of lipid oxidation, kinetically correlated with vesicle permeabilization (Figure 1C). Egg-PC vesicles exhibited a permeabilization extent of about 80% of CF release, in contrast to pure DOPC vesicles, which were little permeabilized by incubation with the same concentrations of iron and ascorbic acid. These results suggest that the polyunsaturated fatty acids present in egg-PC make the lipid bilayers more susceptible to oxidation than the monounsaturated fatty acids present in DOPC or in SM (Figure 1B). The tendency for PUFAs-containing membranes to become more permeable as the degree of unsaturation increases has been previously reported in photosensitized membranes.^29^ Further increases in the concentrations of ascorbic acid and FeSO_4_ were required to permeabilize DOPC vesicles up to 30% of CF release (Figure 1F). Incorporation of saturated SM as well as Chol, two major lipids at the plasma membrane, into DOPC vesicles decreased the permeabilizing activity induced by lipid oxidation, which can be related to the decrease in the proportion of lipids with polyunsaturated fatty acids (Figure 1F).

To characterize the effect of lipid phase coexistence on the sensitivity of LUVs to lipid oxidation, we used vesicles made by the ternary mixture DOPC:SM:Chol (2:2:1), a simple lipid mixture presenting Lo/Ld phase coexistence (Figure 1E) and is commonly used as a minimal model of the outer leaflet of the plasma membrane. Collectively, our experiments show that lipid oxidation induced by Fenton-like reactions causes permeabilization of lipid vesicles of different compositions, confirming that the presence of oxidized lipid species is sufficient to induce membrane permeabilization.

During oxidation, lipid hydroperoxides are initially formed and later truncated to their derivative oxidatively truncated phospholipid species.^35,36^ Structural modifications of oxidized species have been shown to disrupt the regular packing of lipids within the bilayer, which is associated with changes in membrane integrity and permeability.^14,27,36,37^ However, the effects of lipid hydroperoxides and truncated oxidized species on the membrane differ significantly. For instance, lipid hydroperoxides do not necessarily lead to increased membrane permeability.^38^ The anchoring of the hydroperoxide group within the membrane core preserves the barrier function of the membrane although hydration and lateral expansion increase.^39^ In contrast, truncated oxidized lipids can play an important role in membrane permeabilization even at low concentrations.^14^ Their conical shape and ability to induce positive membrane curvature, as well as the tendency of the shorter and highly mobile tail to orient toward the water interface, contribute to the formation of pores and defects in the membrane.^27,40,41^ In this regard, a recent study highlighted the key role of oxidatively truncated phospholipid species in membrane permeabilization during ferroptosis.^9^

### Lipid Oxidation Affects the Lateral Organization of the Lipid Membrane

**Figure 2:**
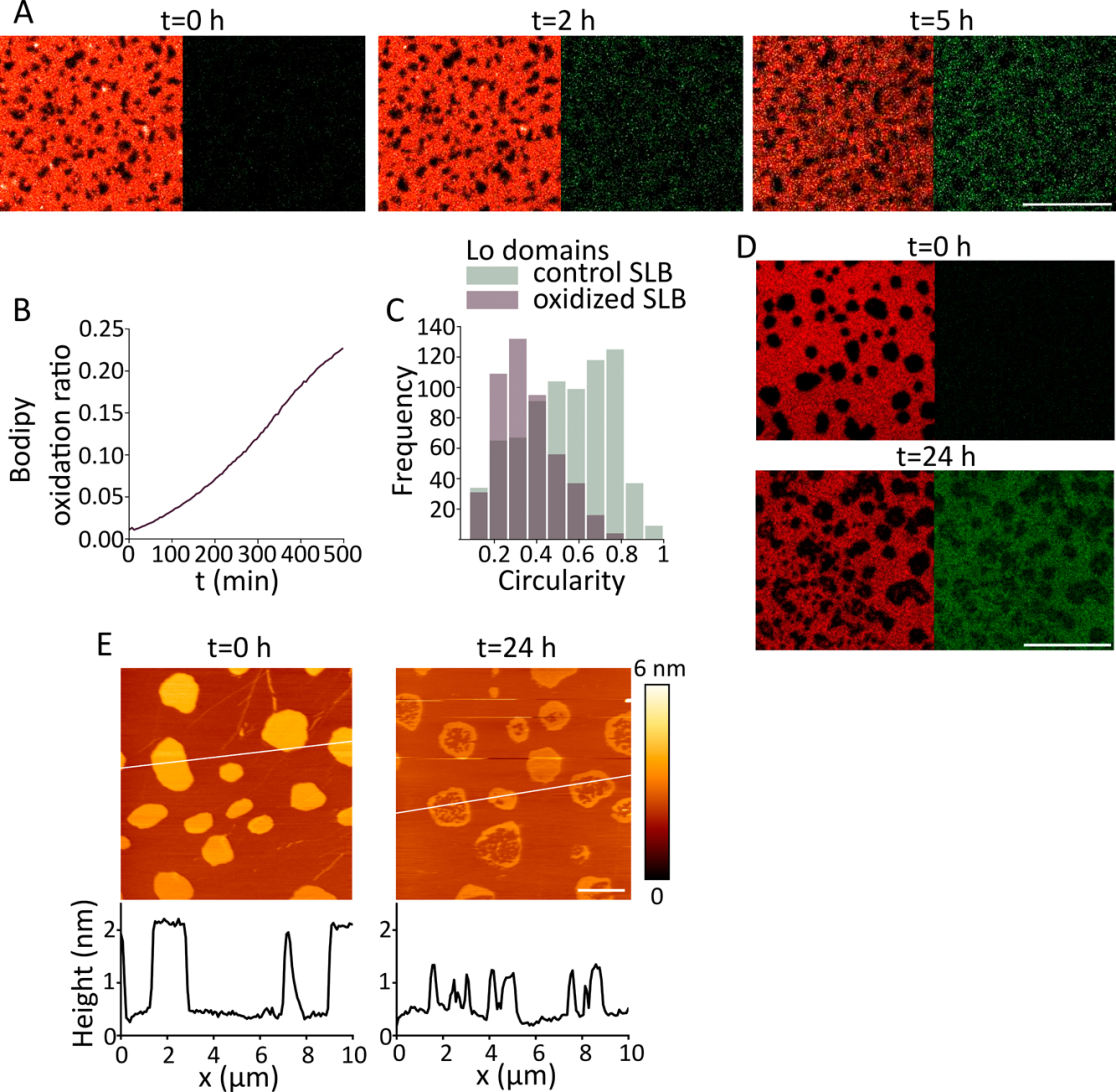
Changes in membrane lateral organization during oxidation. (A) Confocal images of SLBs with composition DOPC:SM:Chol (2:2:1) and labeled with the fluorescent dye BODIPY-C11 (0.1 mol %), before and after 2 and 5 h incubation with 8 mM ascorbic acid and 500 μM iron sulfate at 37 °C. (B) Time course of the increase in the normalized oxidation ratio. (C) Quantification of the circularity of Lo domains in untreated and oxidized SLBs after 24 h of treatment. (D) Corresponding confocal images of SLBs stained with BODIPY-C11 (0.1 mol %) before oxidation and at 24 h, showing changes in domain shape. Scale bars, 10 μm. (E) AFM images of SLBs made of DOPC:SM:Chol (2:2:1) after 5 h oxidation induced with 8 mM ascorbic acid and 500 μM iron sulfate at 37 °C. Corresponding line profiles as indicated by the white line in the AFM image above, *t* = 0 h corresponds to a nonoxidized SLB with Lo and Ld domains. Scale bar, 2 μm.

Lipid oxidation has been observed to shift the miscibility of components in ternary lipid systems, resulting in the appearance of phase coexistence in a previously homogeneous membrane.^13^ To investigate whether lipid oxidation affects the lateral organization of membranes, we visualized supported lipid bilayers (SLBs) made of DOPC:SM:Chol (2:2:1) exhibiting Lo/Ld phase coexistence by confocal imaging and atomic force microscopy (AFM) (Figure 2). Besides the biological relevance of lipid rafts, the round ordered domains that are typical of DOPC:SM:Chol (2:2:1) membranes resulting from line tension at the phase boundary due to phase height mismatch make these membranes a useful tool for studying changes in lateral membrane organization.^42^ To visualize lipid peroxidation in SLBs, we used the lipid peroxidation sensor C11 BODIPY 581/591. In mixtures with Lo/Ld phase coexistence, Lo domains exhibit round shapes that tower over the continuous Ld phase.^21,43−46^ Under these conditions, the hydrophobic tails of the lipids minimize exposure to water by tilting, which causes a line tension at the domain edge.

Since truncated lipid species are secondary products of lipid peroxidation and seem to be the main cause of membrane breakdown,^9,10,25,27^ we decided to study membrane properties at different time points up to 24 h after inducing the formation of oxidized lipid species and their derivatives via iron-mediated Fenton-like reactions to mimic the nonenzymatic oxidation occurring in ferroptotic cells.^5,6^ We found that incubation with iron and ascorbic acid promoted lipid peroxidation at the SLBs, as evidenced by an increase in the green fluorescent signal of BODIPY starting at 5 h (BODIPYox) (Figure 2A,B). Lipid oxidation altered the membrane topography by decreasing the circularity of the Lo domains (Figure 2C,D). The precise characterization of the SLBs using AFM additionally revealed a decrease in the lipid mismatch height between domains at 24 h of lipid oxidation (Figure 2E). These two changes indicate a time-dependent decrease in the line tension at the phase boundaries resulting from the appearance of oxidized lipid species in the membrane. Interestingly, we also detected the appearance of a lower phase with a thickness similar to the Ld phase within the Lo domains, suggesting a reduction in the average lipid order in the SLBs (Figure 2E). It is possible that the increased polarity of lipid hydroperoxides, and truncated lipids and the released acyl fragments, affect their interactions with neighboring lipid molecules, potentially compensating for lipid packing defects at the phase boundaries and thereby affecting the overall organization of the membrane.

Recent studies have investigated the effect of lipid oxidation induced by Fenton reactions using model membranes derived from the plasma membrane.^22^ The authors reported an increased phase demixing, which was attributed to the accumulation of oxidized lipids in the Ld phase, which presented increased area and decreased lipid packing. In this sense, our results are in line with the findings of Kennworthy and colleagues in that lipid disorder and Ld area increase upon lipid oxidation induced by Fenton-like reactions.^22^ Interestingly, while our fluorescence confocal images do not show a disappearance of Lo domains, we visualize a reduced thickness in Lo domains using AFM, suggesting that membrane dynamics may be altered beyond the partitioning into the separate phases.

### Lipid Oxidation Affects the Mobility of Supported Lipid Bilayer

**Figure 3:**
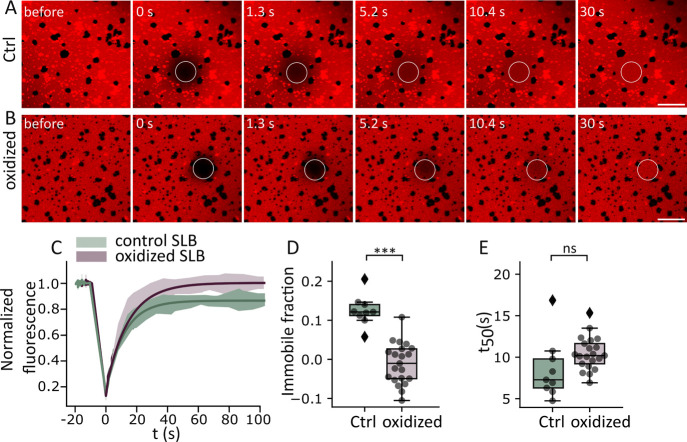
Lipid oxidation decreases the immobile membrane in SLBs. Time-lapse confocal images of a representative FRAP experiment in SLBs made of DOPC:SM:Chol (2:2:1) and labeled with rhodamine-PE (0.5 mol %) in the (A) nonoxidized bilayer and (B) oxidized bilayer after 24 h treatment with 8 mM ascorbic acid and 500 μM iron sulfate. Scale bars, 10 μm. (C) FRAP recovery curves in SLBs before and after lipid oxidation. The graph shows the average (*n* = 9 individual curves in nonoxidized bilayers and *n* = 21 individual curves in oxidized bilayers) temporal increase of the normalized fluorescence. The immobile fraction (D) and *t*_50%_ (E) are calculated by fitting the corresponding recovery curves to an exponential function. Each dot represents a single measurement. In box plots (D and E), the line inside the box indicates the median, the box indicates the interquartile range (IQR) of the values, and the error bars indicate the 1.5 IQR. Statistical comparison between groups was performed by Student’s *t* test, *** indicates independent groups with significant differences *p* < 0.001.

To further study the effect of lipid oxidation on membrane properties, we performed fluorescence recovery after photobleaching (FRAP) experiments using Rhodamine PE as a fluorescent marker, which tends to accumulate in the Ld phase (Figure 3). In nonoxidized bilayers, measurements were restricted to the Ld phase, whereas in the oxidized bilayer the exclusion of Lo domains in the photobleached area could not be guaranteed due to the small size of the Lo domains (Figure 3B). Interestingly, lipid oxidation caused a decrease in the immobile membrane fraction compared to nonoxidized bilayers (Figure 3). Although no significant changes in the *t*_50_ values were observed, the reduction in the immobile fraction suggests that lipid oxidation causes a reorganization of the lipid bilayer into a less compartmentalized environment with increased mobility of the lipid components. This increase in lipid mobility in the oxidized bilayer may be due to the disruption of domain formation as observed by confocal microscopy and AFM (Figure 2). These results are consistent with previous model membrane studies, where incorporation of 10 mol % oxidized phospholipids altered mobility and diffusion.^15^ Changes in shape and length of the oxidation products induce a more heterogeneous bilayer and could lead to disruption of the regular packing. Furthermore, molecular dynamics simulations suggested that cholesterol and PazePC can colocalize and form nanodomains.^37^ Under our experimental conditions, the colocalization of oxidized lipids, such as PazePC and cholesterol, could possibly lead to a reduction in the size of the Lo domains, which would decrease the immobile fraction.^47^

### Lipid Oxidation Decreases the Breakthrough Force of the Membrane

**Figure 4:**
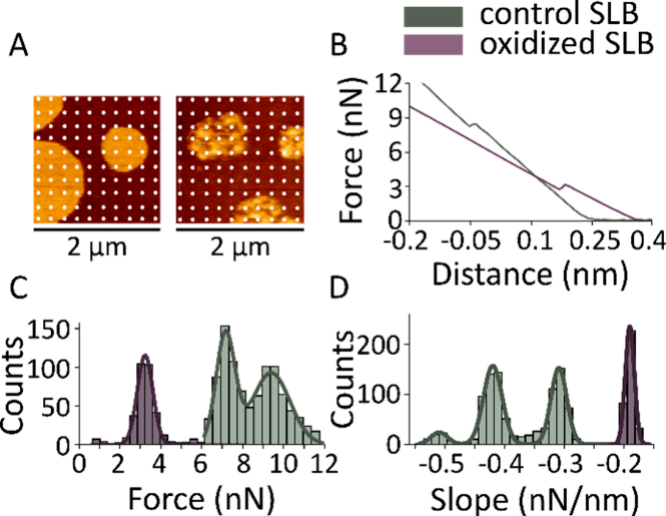
Oxidation decreases the force needed to pierce the membrane. (A) Example grid of force measurements on nonoxidized (left) and oxidized SLBs (right) made of DOPC:SM:Chol (2:2:1) after 24 h treatment with 8 mM ascorbic acid and 500 μM iron sulfate. Each dot represents a position where a force spectroscopy measurement was performed. Images 2 μm × 2 μm. (B) Representative force spectroscopy curves for nonoxidized and oxidized bilayers. Histograms for the distribution of piercing forces (C) and force/distance slopes (D) for nonoxidized (*n* = 954) and oxidized bilayer (*n* = 343). Lines represent the best fit of the histograms data to a bi- and trimodal Gaussian for determination of mean and standard deviation (*R*^2^ > 0.97).

In addition to the high-resolution imaging of SLBs topography, AFM also allows the determination of the physical properties of the membrane in membrane piercing experiments using force spectroscopy (FS). By calibrating the cantilever, its deflection can be used to determine force values. Typical approaching FS curves of oxidized and nonoxidized SLBs made of DOPC:SM:Chol (2:2:1) are shown in Figure 4B. As the AFM tip contacts and pushes against the membrane surface, the force gradually increases until a critical threshold is reached, at which point the membrane is punctured, resulting in a sudden drop in force (breakthrough force).

We found that untreated bilayers exhibit two populations of breakthrough forces around 9.4 ± 0.9 nN and 7.2 ± 0.5 nN, corresponding to the forces required to puncture the liquid-ordered (Lo) and liquid-disordered (Ld) phases, respectively (Figure 4C). After 12 h of incubation with iron and ascorbic acid, we observed that membrane remodeling is accompanied by a significant reduction of the average breakthrough force to 3.2 ± 0.4 nN and the loss of the bimodal behavior, indicating the mixing of lipids from the two liquid phases (Figure 4A,C). Nonoxidized SLBs showed two populations of slope values around −0.31 ± 0.01 nN/nm and −0.42 ± 0.02 nN/nm, whereas the oxidized bilayer showed a single population around −0.19 ± 0.01 nN/nm (Figure 4D). Overall, our results show that the formation of lipid oxidation products via Fenton-like reactions induces strong changes in the mechanical properties of the membrane, leading to a decrease in membrane tension in correlation with the permeabilized state of the bilayer. The relationships between Young’s modulus, force and penetration depth^48^ allow us to compare the slope values and further conclude that oxidized membranes present decreased rigidity. The loss of bimodal behavior in both the breakthrough forces and the slopes of the force–distance curves strongly suggests that oxidized lipid species induce alterations in mechanical properties that are broadly distributed throughout the bilayer.

## Conclusions

Here, we investigated the temporal evolution of the membrane alterations caused by the formation of lipid oxidation products onsite by Fenton-like reactions in pure lipid model membrane systems. In summary, we found here that the formation of lipid oxidation products induced onsite by Fenton-like reactions correlates in time with the increase in liposome permeability, which is associated with higher membrane mobility and with the reorganization of lipid domains, leading to a reduced homogeneity in the liquid order phase domains and a height mismatch between the two phases. We also detected temporal changes in membrane mechanics toward reduced tension in the permeabilized state. We propose that oxidized lipid species, by altering lipid packing, reduce the high energetic cost of pore formation in the lipid bilayer, which may be of relevance in the context of ferroptosis. Pore opening would then allow water entry and further affects the mechanical properties of the bilayer prior to membrane rupture.
